# Sausage Quality during Storage under the Partial Substitution of Meat with Fermented Oyster Mushrooms

**DOI:** 10.3390/foods13132115

**Published:** 2024-07-02

**Authors:** Meltem Boylu, Géza Hitka, György Kenesei

**Affiliations:** 1 Department of Livestock Products and Food Preservation Technology, Institute of Food Science and Technology, Hungarian University of Agriculture and Life Sciences, Ménesi út 43-45, 1118 Budapest, Hungary; kenesei.gyorgy@uni-mate.hu; 2 Department of Postharvest, Commerce, Supply Chain and Sensory Science, Institute of Food Science and Technology, Hungarian University of Agriculture and Life Sciences, Ménesi út 43-45, 1118 Budapest, Hungary; hitka.geza@uni-mate.hu

**Keywords:** meat substitution, meat replacement, sausage, oyster mushroom, fermentation, pretreatment

## Abstract

The increasing global demand for meat production, driven by a rapidly expanding population and changing dietary preferences has prompted the search for protein-rich, sustainable, and healthier meat alternatives. In this context, edible mushrooms are viewed as advantageous substitutes for meat, offering a viable solution. This study aimed to investigate the effects of partially replacing (25% and 50%) pork meat in sausage samples with fermented oyster mushrooms (*Pleurotus ostreatus*), which were subjected to various pretreatments. Six different pretreatments were applied to fresh oyster mushrooms as follows: blanching in water, steaming, oven-cooking, microwave treatment, high hydrostatic pressure treatment, and ultraviolet light treatment. The effects of mushroom replacement on the moisture, pH, lipid oxidation, color, and textural properties of sausages during the 4-week refrigerated storage period were evaluated. The results revealed that replacing pork meat with fermented oyster mushrooms resulted in an increase in moisture content and b* values and a decrease in pH, L*, a*, and shear force values, proportional to the mushroom percentage. The lipid oxidation findings suggest that the antioxidant capabilities of fermented oyster mushrooms were influenced by the pretreatment methods applied to the mushrooms, exhibiting varying levels of effectiveness.

## 1. Introduction

Meat products hold significant dietary prominence for a majority of individuals, owing to their appealing sensory characteristics and valuable nutritional profiles, especially high-quality proteins. However, concerns linked to meat production, such as environmental impact and animal welfare, and health risks stemming from overconsumption are escalating worldwide [1]. Hence, an increasing number of consumers in Western nations are inclined to substitute meat with alternative sources of dietary proteins, at least to some extent, often adopting a Flexitarian diet [2]. In response, the food industry is adjusting current products and/or developing new ones by replacing a part of the meat in the formulations with functional health-promoting and more sustainable components, such as mushrooms [3,4]. Proteins found in edible mushrooms have demonstrated notable biological activities that can enhance health and potentially treat or prevent diseases. Various proteins with significant biological functions have been identified and extracted from edible mushrooms, including lectins, fungal immunomodulatory proteins, ribosome-inactivating proteins, antimicrobial and antifungal proteins, ribonucleases, and laccases. Additionally, bioactive peptides derived from mushrooms have been linked to beneficial health effects [5].

Oyster mushrooms (*Pleurotus ostreatus*), commonly referred to as the “poor man’s meat”, are widely consumed edible fungi. They contain all nine essential amino acids, with leucine, aspartic acid, phenylalanine, and lysine being the most abundant. Oyster mushrooms are also rich in bioactive compounds such as β-glucans, dietary fibers, various vitamins (B1, B2, B12, C, D, and E), phenolic compounds, and carbohydrates [6,7]. Besides being low in fat, calories, and cholesterol, another benefit of substituting meat with oyster mushrooms is their compatibility with meat products, attributed to their umami flavor and fibrous, meat-like characteristics [3]. Several reviews have compiled data on the utilization of various mushrooms as food ingredients and substitutes for meat, fat, salt, and other additives across a wide range of meat products [7,8,9,10]. While numerous studies have investigated the effects of different processing methods on the quality attributes of mushroom varieties [11,12], mushrooms are typically incorporated into meat products in fresh ground form, dried and powdered, or as extracts. However, the adoption of fermentation processes for the production of meat alternatives is still uncommon, despite potential beneficial effects such as generating pleasant aromas and enhancing functionality and nutritional content [13]. Some studies have explored the potential of using cooked oyster mushrooms as an alternative ingredient in a traditional fermented Thai sausage [14] and dried oyster mushrooms for the partial replacement of Pangasius scrapped muscle in a dry-fermented sausage [15]. In these studies, fermentation was employed as the final step in sausage production, but it was not applied directly to the mushrooms themselves as alternative sausage ingredients.

Building on previous research on the pretreatment and fermentation of oyster mushrooms [14], the aim of the current study was to investigate the partial substitution of meat with pretreated fermented oyster mushrooms in sausage formulations and to examine the resulting product characteristics over a 4-week storage period. To date, no studies have been conducted on the utilization of fermented oyster mushrooms as a meat substitute in pork sausages.

## 2. Materials and Methods

### 2.1. Pretreatments, Fermentation, and Sausage Production

**Figure 1:**
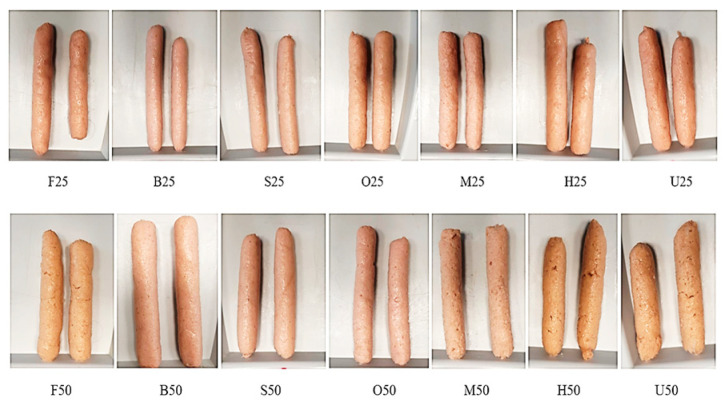
Images of the sausage samples with 25% and 50% meat replacement (F25–F50: sausage with 25–50% fresh fermented oyster mushrooms; B25–B50: sausage with 25–50% blanched fermented oyster mushrooms; S25–S50: sausage with 25–50% steamed fermented oyster mushrooms; O25–O50: sausage with 25–50% oven-treated fermented oyster mushrooms; M25–M50: sausage with 25–50% microwave-treated fermented oyster mushrooms; H25–H50: sausage with 25–50% HHP-treated fermented oyster mushrooms; U25–U50: sausage with 25–50% UV-treated fermented oyster mushrooms).

The oyster mushrooms (*P. ostreatus*) and ground pork (30% fat) used in this study were sourced from a regional market in Budapest, Hungary. Pretreatments and sausage production were performed at the pilot plant of the Hungarian University of Agriculture and Life Sciences, Department of Livestock Products and Food Preservation Technology. After removing undesirable parts, oyster mushrooms were thoroughly cleaned, and sliced lengthwise for pretreatment and fermentation purposes. In addition to fresh oyster mushrooms (F), six different pretreatments [blanching in water (B), steaming (S), oven cooking (O), microwave (M), high hydrostatic pressure (H), and ultraviolet light (U)] were performed along with mushroom fermentation, as described in a previous study [16]. For blanched samples, fresh oyster mushrooms were blanched in boiling water at 100 °C for 3 min. The steaming and oven pretreatments were conducted in an oven (Lainox VE051P, Lainox, Vittorio Veneto, Italy) at 100 °C for 3 min using the steam or oven cooking function. The microwave treatment was applied for 3 min at 85 °C and 100 W using the A3 (vegetable) mode in a microwave oven (SHARP R722STWE, Sharp Electronics Europe Ltd., Middlesex, UK). The HHP treatment was performed on fresh oyster mushrooms at 20 °C and 300 MPa with a 3 min holding time (Resato B2441, Resato International B.V., Assen, The Netherlands). For UV light treatment, fresh oyster mushrooms were exposed to a 312 nm 30 W UV light source (VL-115.M, Vilber Lourmat, Marne La Vallee, France) for 15 min at 20 °C. The mushrooms were placed on a shelf without packaging, with a UV light source positioned 20 cm above them to ensure uniform light irradiation. Following the pretreatments, the mushrooms underwent spontaneous fermentation for 8 days at 21–22 °C. This fermentation occurred in sealed pouches containing 2% (*w*/*w*) salt, 1% (*w*/*w*) sucrose, and 70 mL of 2% salt solution. After the fermentation process, the pouches were unsealed, and the mushrooms were drained to remove excess water. Sausage blends were prepared by combining meat, fermented oyster mushrooms, sodium nitrate, sodium ascorbate, phosphate, and ice in a cutter (Robot-Coupe R202, Robot-Coupe, Burgundy, France). Fifteen different sausage formulations were created using six different pretreatments and three mushroom ratios (0%, 25%, and 50%) replacing meat. The sample groups are presented in Table 1. The sausage blends were filled into 30 mm caliber synthetic casings (NaloShape, ViskoTeepak, Lommel, Germany) using a manual sausage filling machine. The filled sausages were then subjected to a 30-minute thermal process at 80 °C using the steam function of the oven (Lainox VE051P, Lainox, Vittorio Veneto, Italy) and were promptly cooled to below 5 °C. Images of the sausage samples are shown in Figure 1. The moisture content, pH, color, texture characteristics, and lipid oxidation of the sausage samples were assessed on the production day and at intervals of the 7th, 14th, 21st, and 28th days of storage. Three repetitions were conducted for each processing treatment.

### 2.2. Moisture Content and pH

The moisture contents of sausage samples were evaluated following the guidelines outlined in the AOAC 950.46 method [17]. For the calculations, 3 g of sausage samples were dehydrated using a convection oven (Labor Müszeripari Müvek, Budapest, Hungary) at 105 °C for 16 h. The pH levels of the sausage samples were measured using a calibrated pH meter designed for meat products (Testo SE, Titisee-Neustadt, Germany). The pH electrode was directly inserted into the samples, and the measurements were taken across three separate trials.

### 2.3. Color Measurement

The color properties of the sausage samples were evaluated using the CIELAB scoring system [18]. The lightness (L*), redness (a*), and yellowness (b*) of the sausage samples were assessed with an 8 mm aperture size CR-410 colorimeter (Konika Minolta Sensing Inc., Osaka, Japan), employing a 2° observer and calibrated with illuminant C on a standard white reflectance calibration plate (CRA43). Measurements were taken at room temperature from the freshly cut surfaces of the samples. Each sausage sample underwent nine parallel measurements.

### 2.4. Texture Properties

The TA.XT Plus texture analyzer (Stable Micro System, Surrey, UK) was used to evaluate the textural properties of the sausage samples. Shear force measurements were conducted by cutting through the sausage samples using a Warner–Bratzler shear blade at a velocity of 2 mm/s and a set distance of 30 mm. The shear force value was determined as the maximum peak force (N) recorded during each measurement, as a function of distance. The maximal force (Fmax, N) served as the texture parameter to assess the hardness of the samples. Nine measurements were conducted for each sausage sample.

### 2.5. Lipid Oxidation

The Thiobarbituric Acid Reactive Substances (TBARS) values in sausages were determined as an indicator of lipid oxidation during storage, following the Tarladgis method [19]. A 5 g sausage sample was homogenized with 20 mL of 5% trichloroacetic acid (TCA) for 2 min using a Digital Ultra-Turrax (Staufen, Germany) and centrifuged at 4500 rpm and 4 °C for 10 min. Supernatants were filtered through Whatman No. 1 filter paper, and 2 mL of the filtrate was mixed with 2 mL of thiobarbituric acid (TBA 0.08% *w*/*v*) in a glass tube. These tubes were then placed in a water bath at 95 °C for 30 min and subsequently cooled to ambient temperature. Absorbance readings were taken at 532 nm against a blank solution (a mixture of 2 mL of 5% TCA and 2 mL of 0.08% TBA) using a U 2900 UV–visible spectrophotometer (Hitachi Ltd., Tokyo, Japan). The TBARS values were expressed as milligrams of malondialdehyde (MDA equivalent) per kilogram of sausage samples.

### 2.6. Statistical Analysis

The acquired data were analyzed using one-way multivariate analysis of variance (ANOVA) in IBM SPSS software (Version 29, SPSS Inc., Chicago, IL, USA). Homogeneity across the measured values was ensured through Levene’s test. Tukey’s post hoc analyses were performed to discern any notable distinctions among the samples. The observed differences were considered statistically significant at a significance level of *p* < 0.05.

## 3. Results and Discussion

### 3.1. Moisture Content

The moisture contents of the sausage samples with 25% and 50% meat replacement with fermented oyster mushrooms are given in Table 2. Significant differences were observed in the moisture content of the sausage samples (*p* < 0.05). All meat replacement samples had higher moisture contents compared with the C sample, and the 50% meat replacement samples exhibited higher moisture content compared with both the C and 25% replacement samples. This can be attributed to the fact that the fermented mushrooms that replaced meat had a higher (85–90%) moisture content [16]. Similar results were reported when oyster mushrooms were used to replace meat in sausages, where the control sample had the lowest moisture content (67.62 ± 0.33%) and sausages incorporating 60% fresh oyster mushrooms (75.29 ± 1.44%) had the highest [20].

Among the samples with 25% meat replacement, the U25 samples had the lowest moisture content (62.04 ± 0.10%) on day 0, similar to the control samples (62.35 ± 0.11%). However, from day 0 to day 7, the moisture content of the C samples significantly decreased (56.32 ± 0.56%) and remained the lowest throughout the storage period, while the other samples showed an increasing trend in general. The highest moisture content on day 0 was observed in the B25 samples (67.82 ± 0.12%) and remained the highest throughout the storage period, reaching 70.36 ± 0.26% by the end.

The moisture content of the sausage samples with 50% meat replacement also showed significant differences. Similar to the 25% replacement case, the U50 samples had the lowest moisture content on day 0 (71.10 ± 0.38%) and also on day 28 (70.00 ± 0.11%). Except for the S50 sample group, the moisture content of all the samples exhibited fluctuations over the course of storage. The moisture content of the B50 samples (75.55 ± 0.16%) measured the highest at the end of the storage period, followed by the S50 samples (75.37 ± 0.24%).

The higher moisture contents observed in the B25 and B50 samples compared with the other samples could be attributed to the moisture-retaining properties of the blanching pretreatment. Blanching helps seal moisture within mushroom tissues, thereby preserving their water content before fermentation [21]. Consequently, when incorporated into sausage formulations, they may contribute more moisture to the final product than the mushrooms subjected to the other pretreatments. The lowest moisture content in U25 and U50 compared with the other sample groups, could possibly be linked to surface dehydration effects caused by UV exposure, as suggested by other researchers [22]. However, this effect was not observed in our prior research. Other authors also reported differences in the moisture content of pork sausages with *Pleurotus eryngii* subjected to different treatments [23]. Variations in microbial activity during fermentation influenced by different pretreatments and subsequent incorporation could also contribute to these differences in the moisture contents of the sausage samples.

### 3.2. pH

**Figure 2:**
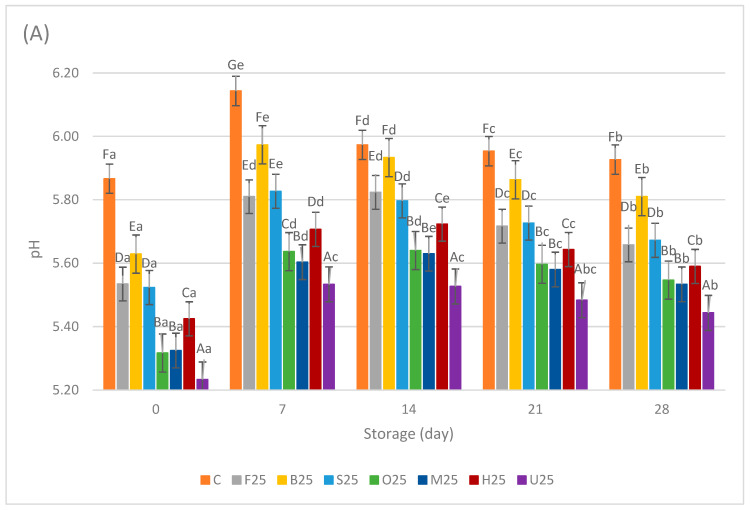

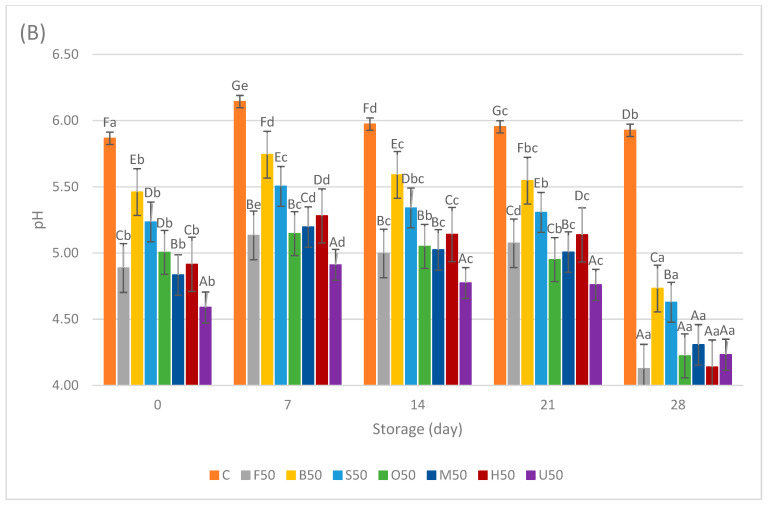
Means ± standard deviations of pH levels of sausage samples during storage with 25% meat replacement (**A**) and with 50% meat replacement (**B**). ^A–G^; Different letters indicate significant differences among sample groups. ^a–e^ Different letters indicate significant differences among storage days (Tukey’s post hoc test, *p* < 0.05).

The pH values of the sausage samples are given in Figure 2A,B. The meat replacement with pretreated fermented mushrooms significantly affected the pH of the sausage samples (*p* < 0.05). The control samples exhibited higher pH values compared with all other replacement samples starting from day 0 (pH = 5.87 ± 0.03) through the end of the storage period (pH = 6.04 ± 0.02). This difference was expected because of the lower pH values of the fermented mushrooms compared with the replaced meat.

Among the 25% replacement samples, B25 exhibited the highest pH value starting from day 0 (pH = 5.63 ± 0.01) and continuing until day 28 (pH = 5.81 ± 0.01), whereas the U25 samples exhibited the lowest from day 0 (pH = 5.23 ± 0.03) until the end of the storage period (pH = 5.44 ± 0.01). All sample groups displayed a trend of increasing pH until day 7, followed by a subsequent decrease until the end of the storage period, except for the control samples.

A similar order was observed among the 50% replacement samples, which exhibited lower pH values compared with their 25% replacement counterparts, ranging from 4.58 ± 0.01 to 5.46 ± 0.01 after production. There was a slow decreasing trend in the pH values of samples from day 7 to day 21. However, a notable decline in pH values was observed from day 21 to day 28. At the end of the storage period, there were no significant differences among the F50, O50, M50, H50, and U50 samples, all exhibiting a low pH level close to 4.3. This outcome could be attributed to the prior research findings, where fresh, oven-cooked, HHP-treated, and UV-treated fermented mushroom samples had lower pH values compared with blanched and steamed fermented mushroom samples [16]. Similar results were reported with *Agaricus bisporus* mushrooms, where boiled mushrooms had a higher pH value than sous-vide cooked, pan-fried, oven-cooked, and barbecued mushrooms. This phenomenon was hypothesized to stem from prolonged exposure of the samples to hot water during pretreatments such as blanching and steaming, leading to thermal degradation of organic acids, their leakage into water, and consequent reduction in acidity [12]. In contrast, pretreatments involving UV and HHP do not induce the breakdown of organic acids through heat, and microwave pretreatment involves heating internally and unevenly, potentially preserving more of the natural acidity compared with conventional thermal methods. In a study on a vegan boiled sausage analog, the pH of the samples with *Pleurotus sapidus* was measured as 4.7 ± 0.01 on the production day and 4.6 ± 0.02 after 4 weeks of storage. The authors noted that a pH decrease occurred over time not only in meat products but also in vegetarian/vegan systems containing fungal mycelia because various microorganisms metabolize sugars and proteins, leading to increased concentrations of organic acids [24]. In another study on fermented sausages based on meat substitutes from soybean protein and the *Coprinus comatus* mushroom, a pH value of 5.09 was reported within 18 h [25]. On the other hand, when dry *Lentinula edodes* was used as pork meat replacer in sausages, higher pH values were observed compared with this study, with values of 6.65 ± 0.03 and 6.69 ± 0.03 for 25% and 50% replacement samples, respectively [26].

### 3.3. Lipid Oxidation

**Figure 3:**
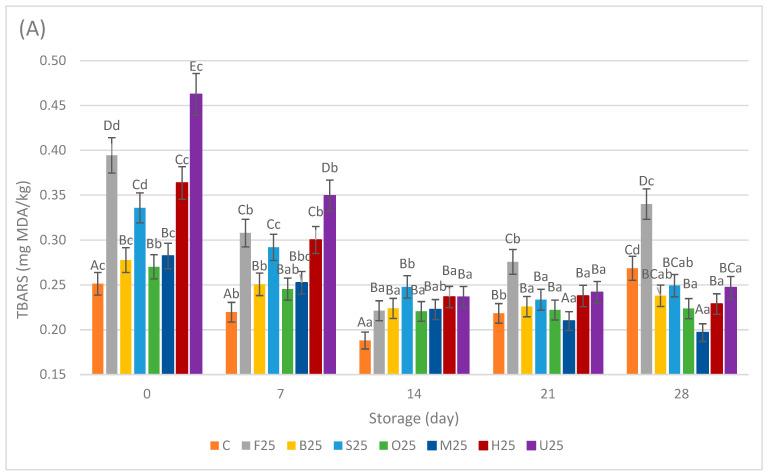

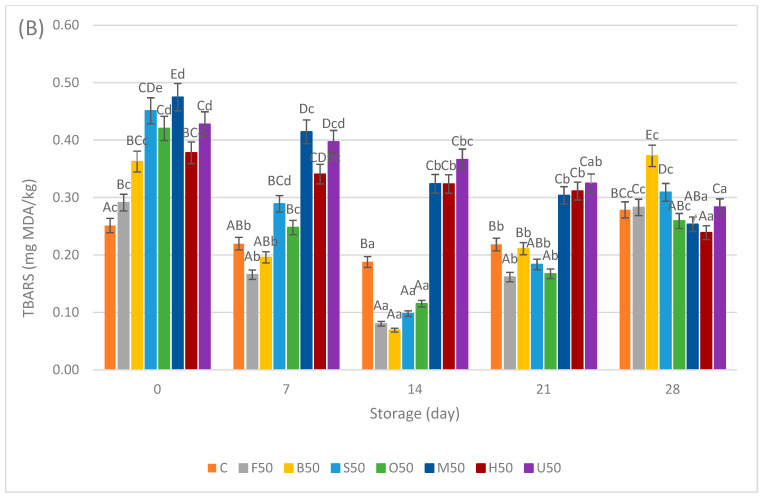
Means ± standard deviations of the TBARS levels of the sausage samples during storage with 25% meat replacement (**A**) and 50% meat replacement (**B**). ^A–E^; Different letters indicate significant differences among sample groups. ^a–e^ Different letters indicate significant differences among storage days (Tukey’s post hoc test, *p* < 0.05).

The results of the TBARS-measured malondialdehyde levels of the sausage samples during storage are given in Figure 3A,B. The meat replacement with pretreated fermented mushrooms significantly affected the TBARS values of the sausage samples (*p* < 0.05). Among the 25% replacement samples, the TBARS values ranged from 0.27 ± 0.01 to 0.46 ± 0.01 mg MDA/kg on day 0. The control samples consistently exhibited lower TBARS values compared with the samples with 25% replacement from day 0 (0.25 ± 0.00 mg MDA/kg) to day 14 (0.19 ± 0.01 mg MDA/kg). However, the TBARS values of both the C and F25 samples increased after day 14, while other sample groups remained relatively stable within the range of 0.20–0.25 mg MDA/kg. By the end of the storage period, both the C and F25 samples returned to similar TBARS values as on day 0. The remaining sample groups (B25, S25, O25, M25, H25, U25) exhibited a significant decrease in TBARS values at the end of the storage period compared with their initial values (*p* < 0.05). On day 28, the highest TBARS value was observed in F25 (0.37 ± 0.03 mg MDA/kg), while the lowest was measured in the M25 (0.20 ± 0.02 mg MDA/kg) samples. 

Among the 50% replacement samples, the TBARS values ranged from 0.29 ± 0.01 to 0.50 ± 0.01 mg MDA/kg on day 0. Although the TBARS values decreased across all sample groups by day 14, there was a distinct classification into three categories observed among the sample groups at this time. The TBARS values of the M50, H50, and U50 samples (I) were observed to be significantly higher than those of the C (II), F50, B50, S50, and O50 (III) samples. This categorization could be attributed to the distinctive nature of the various pretreatment methods applied. The antioxidant capacity of mushrooms and, consequently, the lipid oxidation of sausages may be affected differently by novel techniques such as microwave, HHP, and UV light pretreatments compared with traditional heat pretreatments. A significant reduction in total antioxidant activity was reported with microwave blanching for 3 min at 85 °C, and better outcomes regarding temperature distribution, polyphenol oxidase inactivation, weight loss, shrinkage, total antioxidant activity, and sample browning were achieved using a combined method (1 min in the microwave at 85 °C followed by a 20-s water bath at 92 °C) [27]. For instance, UV stress is recognized to cause harm to plant tissues by triggering oxidative stress, resulting in lipid peroxidation, protein denaturation, carbohydrate oxidation, and DNA damage [28]. Researchers concur that pressure treatments (HHP) often prompt oxidation and have proposed several hypotheses, including the release of iron from heme and conformational alterations in hemoproteins under pressure, leading to increased exposure of unsaturated fatty acids to catalytic heme groups [29,30]. In a study on pork samples, it was reported that samples exhibited more secondary oxidation products and higher TBARS values as the pressure level of HHP treatments increased [31]. Conversely, traditional heat pretreatments such as blanching, steaming, and oven cooking are types of thermal processing that can result in the formation of new compounds with antioxidant properties, such as those produced by the Maillard reaction [32]. In one study, it was reported that the phenolic content of *Pleurotus ostreatus* was negatively affected by the blanching and fermentation processes, possibly because of the diffusion of soluble phenolics into the fermentation brine [11]. In this study, towards the end of the storage period, these differences among the TBARS values of the samples mostly diminished. The notable decrease in TBARS values of all samples on day 14 could be attributed to an initial phase of oxidative stress in the samples after processing, leading to elevated TBARS values at the beginning of the storage period. As this stress diminishes and the product stabilizes over time, TBARS values tend to decline, as seen on day 14 [33]. On the other hand, a study found that Porcini mushrooms (*Boletus edulis*) added to frankfurters showed a peak in antioxidant activity around day 20 of cold storage. However, this activity decreased thereafter because of the depletion of the mushrooms’ natural antioxidant reserves, as observed in this study after day 14 [34]. Regarding the TBARS data, it is noteworthy that all sample groups displayed lipid oxidation levels below 0.50 mg MDA/kg of the sample during the whole storage period, which falls within the acceptable range for processed meat products [35]. This result implies that the antioxidant properties of fermented oyster mushrooms, as reported before [36], can maintain a certain level of effectiveness when incorporated into sausage products. These antioxidant properties are affected by the pretreatment methods used on the mushrooms. Additionally, a consistent decrease was observed in TBARS values for the M, H, and U samples throughout the storage period, despite starting with higher initial TBARS values. In another study where dry *Lentinula edodes* was used to substitute 25%, 50%, 75%, and 100% of pork meat in sausages, it was reported that the total phenolic content and antioxidant activity of the sausages improved compared with the control samples [26].

### 3.4. Color

**Figure 4:**
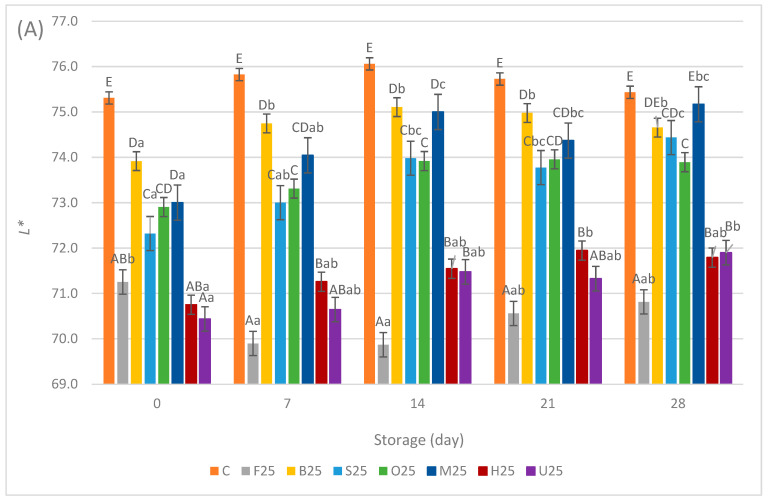

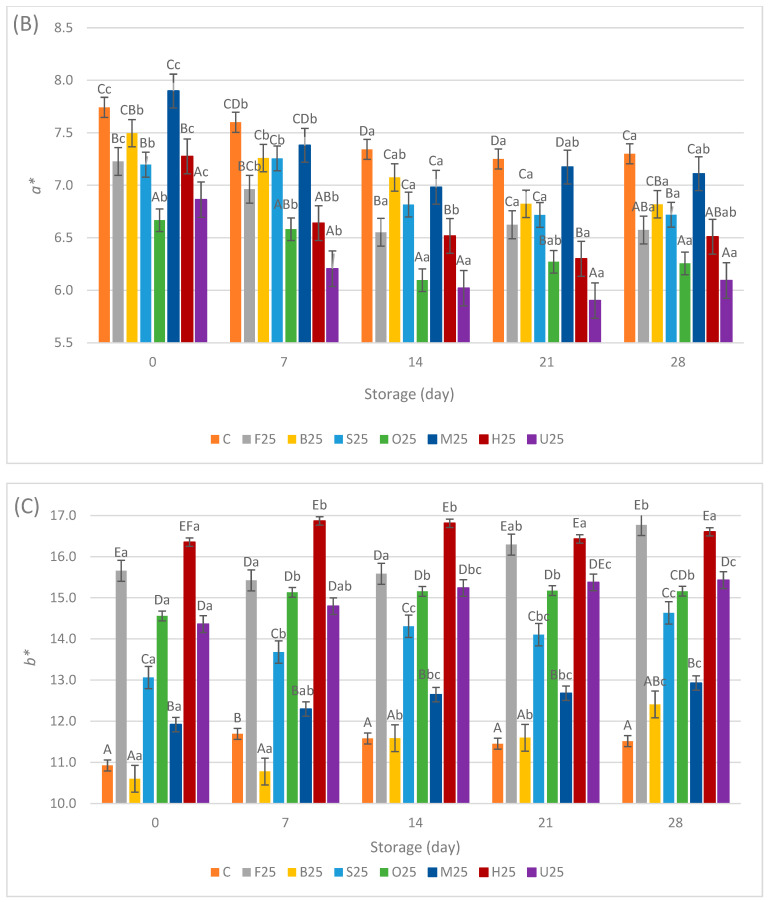
Means ± standard deviations of the color attributes (**A**) L*, (**B**) a*, and (**C**) b* of sausage samples during storage with 25% meat replacement. ^A–F^; Different letters indicate significant differences among sample groups. ^a–c^; Different letters indicate significant differences among storage days (Tukey’s post hoc test, *p* < 0.05).

**Figure 5:**
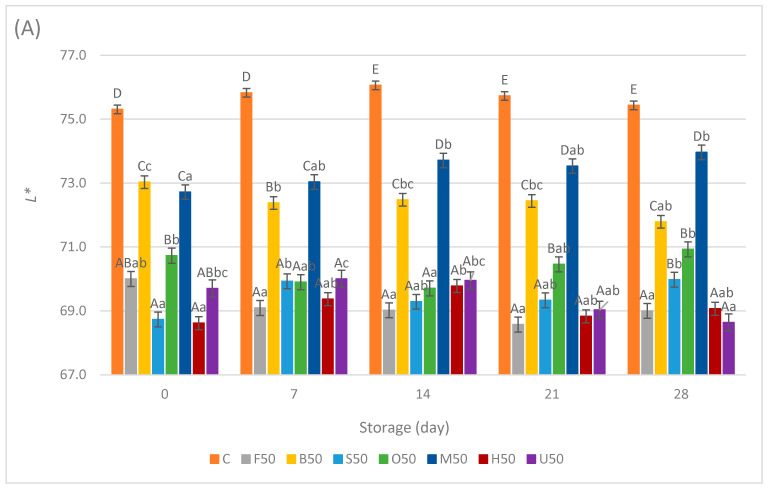

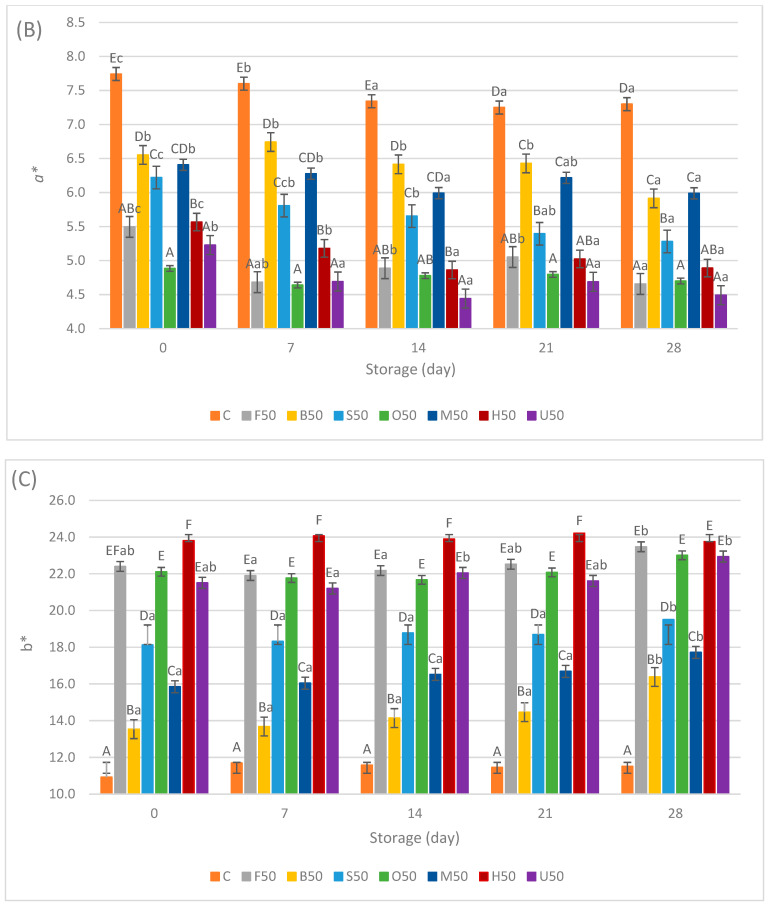
Means ± standard deviations of color attributes (**A**) L*, (**B**) a*, and (**C**) b* of sausage samples during storage with 50% meat replacement. ^A–F^; Different letters indicate significant differences among sample groups. ^a–c^; Different letters indicate significant differences among storage days (Tukey’s post hoc test, *p* < 0.05).

The instrumental color evaluation (L*, a*, b*) of the sausage samples during the storage period is shown in Figure 4A–C and Figure 5A–C. Significant differences were observed among the samples regarding color attributes (*p* < 0.05). The L* values of the C samples consistently remained higher on all storage days examined, compared with the samples both with 25% and 50% replacement. There were no significant changes in the L* values of the C samples starting from 75.31 ± 1.45 on day 0 to 75.43 ± 0.7 on day 28. 

Throughout the storage period, the F25 samples consistently exhibited the lowest L* values, ranging from 69.87 ± 1.08 to 71.25 ± 0.5, followed by the H25 and U25 samples. These samples stood distinct from the S25, O25, M25, and B25 samples, which displayed significantly higher L* values. Furthermore, the lightness increased to varying extents for all the 25% replacement samples except F25, suggesting a consistent trend of slight brightening over time across these products, probably because of the oxidation of fat.

A similar order was observed in the samples with 50% replacement, where the M50 and B50 samples exhibited higher L* values than others. The F50, S50, H50, and U50 samples displayed the lowest L* values with slight differences, ranging from 68.63 ± 0.45 to 69.97 ± 0.68 throughout the storage period. In terms of sausage sample lightness, it can be inferred that replacing meat with fermented oyster mushrooms resulted in a slight darkening of the sausage color, with variations depending on the pretreatment method applied. This effect was slighter for the sausage samples when blanching or microwave pretreatments were applied. Previous studies have documented similar effects of blanching and microwave treatments on the color properties of canned mushrooms, leading to similar enzymatic inactivation and structural changes within the cells, which minimally affect the mushrooms’ pigment composition and retention, thus resulting in similar color outcomes [37]. On the other hand, the darkening effect of the HHP treatment in this study can be attributed to the activity of polyphenol oxidase at 300 MPa. Polyphenol oxidase, particularly Tyrosinase, is an enzyme within the polyphenol oxidase family that serves as a primary catalyst for the browning of *A. bisporus* and *P. ostreatus* because of its high concentration. [38]. When polyphenol oxidase inactivation was not accomplished, the enhanced permeabilization of cell membranes due to phospholipid crystallization allows extracellular polyphenol oxidase to interact with phenols, leading to increased browning during pressure treatment. [39]. An upward trend in L* and b* values was documented in carambola puree as pressure levels escalated from 200 MPa to 600 MPa and 800 MPa. This phenomenon was explained by the enzymatic inactivation of polyphenol oxidase under higher pressure conditions [40,41]. In a study involving oyster mushrooms, extending the UV-C exposure time beyond 30 min resulted in browning. This darkening can be attributed to UV treatment activating polyphenol oxidase, which oxidizes phenolic compounds into quinones. These quinones then polymerize, forming brown pigments and causing the mushrooms to develop a darker color [42]. This effect was observed even though the exposure time in our study was 15 min. These findings align with those of other authors [20], who observed a decrease in sausage lightness from 75.59 ± 0.84 to 72.16 ± 0.63 when reducing chicken meat and increasing fresh oyster mushroom levels up to 45% in their samples. Similar results were also reported with *Lentinula edodes* as a meat replacer in sausages [26] and with *A. bisporus* and *P. ostreatus* flour addition in frankfurter sausages [43].

There was a significant decline in a* values for all sample groups during storage. The a* values of samples with 25% replacement ranged between 5.90 and 7.89 during the storage period. Both the B25 and M25 samples showed similar a* values to those of the C sample (7.30–7.74), which were higher compared with the other sample groups, on all days of examination. The lowest a* values were recorded in the U25 (6.09 ± 0.41) and O25 (6.25 ± 0.47) samples at the end of the storage. A similar trend to the 25% replacement samples was observed in the 50% replacement samples, with the a* values falling within a lower range, from 4.49 to 6.74. In this instance, the C sample was clearly distinct from all the other samples, exhibiting higher a* values during the whole storage period (*p* < 0.05). It is worth noting that substituting 25% of the meat with fermented oyster mushrooms did not result in a loss in red color for the B25 (blanched fermented) or M25 (microwaved fermented) samples. A study of microwave blanching on the quality of frozen *Agaricus bisporus* mushrooms also revealed that the color spectrum of microwave-treated frozen mushrooms shifted more towards red and less towards yellow compared with those blanched with sodium metabisulfite [44]. This could be explained by microwave radiation penetrating the mushroom tissues and affecting the distribution and structure of pigments, potentially altering their absorption and reflection properties. This alteration can enhance the intensity of red hues while reducing the presence of yellow tones, resulting in the observed color shift towards red. However, when the replacement ratio increased to 50%, all sample groups showed a decrease in red compared with the control sample because of the reduced meat content in the formulations. Similar decreases in a* values were observed in frankfurter sausages by the addition of *Agaricus bisporus* and *P. ostreatus* [43] and in goat meat nuggets with *Flammulina Velutipes* mushroom [45]. On the other hand, no difference was observed regarding the a* values of beef patties when button mushrooms were used as meat extenders even up to a 50% ratio [46].

Throughout the storage period, the b* values of samples with 25% replacement exhibited a significant increase (*p* < 0.05). The highest b* values were recorded in the F25 (16.77 ± 0.47) and H25 (16.60 ± 0.31) samples by the end of the storage period. Conversely, the lowest b* values were observed in the B25 (10.60 ± 0.53 to 12.40 ± 0.46) and C (10.92 ± 0.60 to 11.51 ± 0.73) samples. A similar trend was observed in the 50% replacement samples, with higher b* values (13.53 ± 0.44 to 24.21 ± 0.20) compared with their 25% counterparts, maintaining a consistent order among the sample groups. Additionally, when the replacement ratio increased to 50%, all sample groups exhibited a more yellow color compared with the control sample (*p* < 0.05) because of the natural color of mushrooms. The highest b* values were recorded in the H50 (23.75 ± 0.70 to 23.80 ± 0.39) samples on all days of examination. The increased yellowness index observed with pressure treatment was attributed to an increase in beta-carotene content within the carambola puree samples [40]. The color results are in agreement with those of another study [23], reporting that using *Pleurotus eryngii* with different treatments in pork sausages, as replacements for pork back fat, resulted in more yellow sausages. However, in their study, the b* values of the sausages were not significantly affected when the mushrooms were added in their raw form but were affected when boiled, deep-fried, or fried. Color outcomes are substantially influenced by the color of ingredients in the formulation, their species, as well as their respective quantities. Our study revealed that the pretreatments applied to mushrooms before their fermentation resulted in significant differences in the color attributes of the meat-replaced sausage samples. The most adverse effects on the color of the sausages were observed when the mushrooms were fresh, HHP-treated, or UV-treated prior to their fermentation. Conversely, the least adverse effects were observed when the mushrooms were blanched or microwaved prior to fermentation.

### 3.5. Texture

**Figure 6:**
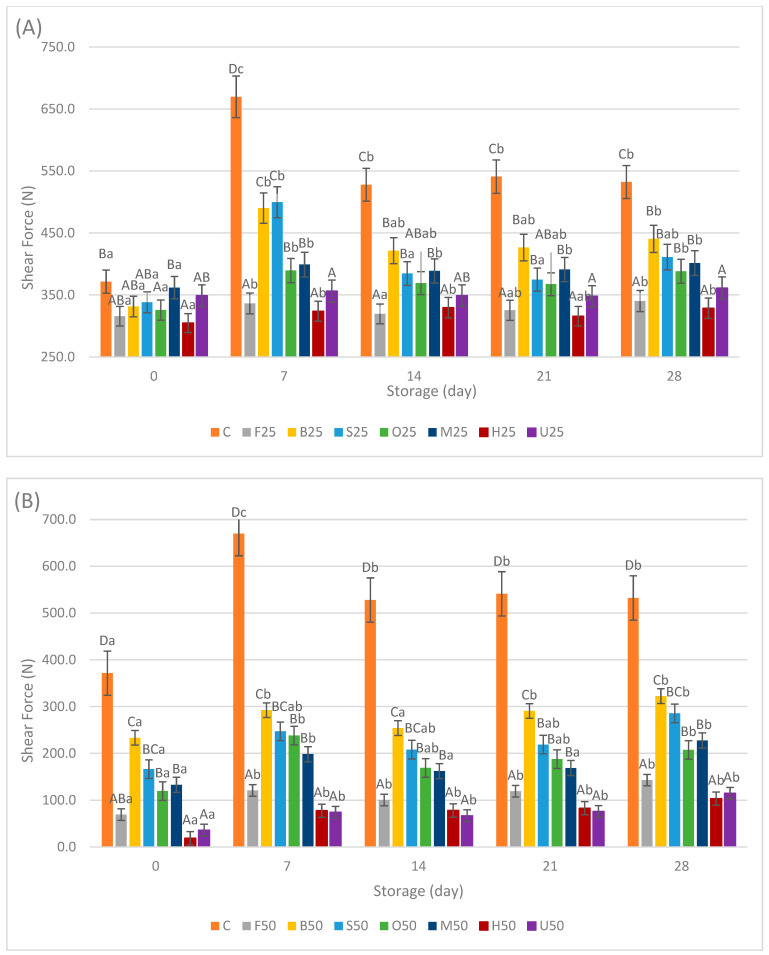
Means ± standard deviations of shear force of sausage samples during storage with 25% meat replacement (**A**) and 50% meat replacement (**B**). ^A–D^; Different letters indicate significant differences among sample groups. ^a–c^; Different letters indicate significant differences among storage days (Tukey’s post hoc test, *p* < 0.05).

The texture evaluation of the sausage samples is demonstrated in Figure 6A,B. Meat replacement with pretreated fermented mushrooms significantly affected the texture of the sausage samples (*p* < 0.05). The control samples consistently showed the highest shear force values compared with all the replacement samples from day 0 (371.50 ± 32.53 N) until the end of the storage period (532.33 ± 29.12 N), indicating greater sausage hardness.

Among the samples with 25% replacement, B25, S25, O25, and M25 exhibited a harder texture compared with the F25, H25, and U25 samples (*p* < 0.05). A notable rise in hardness values was observed for all samples except U25 on day 7. By the end of the storage period, these samples showed higher shear force values compared with their initial measurements (*p* < 0.05).

For the samples with 50% replacement, a comparable pattern was noted, with the B50, S50, O50, and M50 samples showing a harder texture compared with the F50, H50, and U50 samples. However, when the replacement ratio was at 50%, the force values varied between 18.56 ± 5.24 and 322.45 ± 37.36, whereas at the 25% replacement ratio, they ranged from 325.58 ± 14.37 to 499.67 ± 21.23 during the storage period. This suggests that substituting meat with fermented oyster mushrooms and increasing the replacement ratio from 25% to 50% in the sausage formulations led to a softer texture. A significant increase was also observed in force values of all the replacement samples (except U) at the end of the storage period, compared to their initial values. 

Similar results were detected in frankfurters with *Agaricus bisporus* and *P. ostreatus* mushrooms, where cold storage caused an increase in the hardness of samples as a result of purge loss. The same study reported that the addition of *Pleurotus ostreatus* led to softer sausage samples [43]. Another study on emulsion-type sausages reported that replacing chicken breast meat with *Agaricus bisporus* mushrooms at higher rates than 10% resulted in significantly decreased hardness, springiness, cohesiveness, and chewiness compared with those of control sausages. This indicated that mushrooms have a softer texture, which agrees with the lower protein content and higher moisture content compared with chicken breast meat, as sausage hardness was closely related to the protein and water content [47]. In general, softer textures were observed in meat products when various mushrooms were incorporated, a phenomenon attributed to mushroom dietary fibers trapping fluids and a decreased concentration of solubilized muscle proteins. This combination results in a softer texture in final muscle food products [3]. In their study on pork sausages using *Pleurotus eryngii* with different treatments, authors reported that incorporating raw, boiled, and deep-fried *Pleurotus eryngii* resulted in decreased hardness of the sausages, whereas the sausages containing fried *Pleurotus eryngii* exhibited a harder texture [23].

In this study, all the replacement samples demonstrated a softer texture compared with the control samples on all days of examination. However, the most significant alterations were observed in sausage samples where fresh fermented, UV-fermented, and HHP-fermented mushrooms were utilized at both 25% and 50% ratios. The soft texture of fresh fermented samples highlights the significance of pretreatment processes to preserve the quality of mushrooms prior to their fermentation [27]. The softer texture of HHP-treated samples aligns with a study that examined the effects of HPP at levels of 100–300 MPa with holding times of 3–18 min on the quality characteristics of fresh *Pleurotus eryngii.* It was revealed that with increasing pressure and holding time, PPO enzyme activity and sample elasticity initially declined but subsequently increased. Conversely, the L* values and hardness of the samples gradually decreased, while the b* values increased [48,49]. Unlike vegetables and fruits, which possess rigid cell walls for structural support, mushrooms are primarily composed of glucan and chitin. High pressures can rupture their membranes, causing substantial redistribution of water at a microscopic level, alongside the gelatinization of cell biopolymers. When mushrooms are pressurized, the cell proteins denature, causing an increase in permeability. This heightened permeability leads to the loss of cellular solutes and water, ultimately resulting in softened cells [50]. On the other hand, the softer texture observed in UV-treated samples aligns with a study that examined the effects of UV-B pretreatment on mushrooms. It was indicated that UV-B pretreatment reduces the fracture force of the treated samples compared with the control samples. This reduction in fracture force is likely due to the pretreatment shortening drying time and causing damage to cell walls, thus softening the texture of the samples [22]. However, research on UV light treatment of mushrooms primarily focuses on vitamin D content, with limited studies addressing its impact on texture. Different results were reported for pulsed light treatment, which had a dramatic effect on the textural properties of fresh-cut *Agaricus bisporus* mushrooms because of the thermal damage caused by high fluencies [51]. In contrast, white LED irradiation was found to better maintain the textural properties such as firmness, chewiness, and resilience of shiitake mushrooms [52]. These varying results could be attributed to the different types of light, light dose, and the mushroom species used in these studies.

## 4. Conclusions

The utilization of fermented oyster mushrooms as a substitute for meat in the sausage formulations affected all the investigated quality attributes of the samples throughout storage. In general, the 25% replacement samples presented better results than the 50% replacement samples, suggesting that the extent of substitution is limited by the proportion of fermented mushrooms used. Various pretreatment methods applied to mushrooms before their fermentation also had an impact on the quality parameters of the sausages. Novel technologies such as HHP and UV light were found to be unsuitable for the pretreatment of mushrooms because of significant quality alterations. Conversely, microwave pretreatment could be considered a viable alternative to conventional thermal pretreatments. Overall, substituting meat with blanched + fermented, steamed + fermented, and microwave + fermented mushrooms resulted in better results, causing less darkening and a less soft texture of the sausages compared with the fresh + fermented, UV + fermented, and HHP + fermented mushrooms. The results indicate that utilizing fermented oyster mushrooms as a substitute for meat in sausage formulations is feasible but restricted by the proportion of mushrooms and contingent upon the specific pretreatment technology used. Additional research is required to assess the microbiological safety and sensory attributes of the obtained products.

## Figures and Tables

**Table 1:** Sample groups.

Sample	Description
C	Control sample, 100% ground pork sausage
*Sausage samples with 25% meat replacement*	
F25	25% Fresh fermented oyster mushrooms + 75% ground pork
B25	25% Blanched fermented oyster mushrooms + 75% ground pork
S25	25% Steamed fermented oyster mushrooms + 75% ground pork
O25	25% Oven-treated fermented oyster mushrooms + 75% ground pork
M25	25% Microwave-treated fermented oyster mushrooms + 75% ground pork
H25	25% HHP-treated fermented oyster mushrooms + 75% ground pork
U25	25% UV-treated fermented oyster mushrooms + 75% ground pork
*Sausage samples with 50% meat replacement*	
F50	50% Fresh fermented oyster mushrooms + 50% ground pork
B50	50% Blanched fermented oyster mushrooms + 50% ground pork
S50	50% Steamed fermented oyster mushrooms + 50% ground pork
O50	50% Oven-treated fermented oyster mushrooms + 50% ground pork
M50	50% Microwave-treated fermented oyster mushrooms + 50% ground pork
H50	50% HHP-treated fermented oyster mushrooms + 50% ground pork
U50	50% UV-treated fermented oyster mushrooms + 50% ground pork

**Table 2:** Means ± standard deviations of the moisture contents of the meat-replaced sausage samples during storage.

	Moisture Content (%)					
	Storage (Day)					
	Samples	0	7	14	21	28
	C	62.35 ± 0.11 ^Ab^	56.32 ± 0.56 ^Aa^	55.94 ± 0.29 ^Aa^	56.11 ± 0.20 ^Aa^	56.44 ± 0.09 ^Aa^
25% meat replacement	F25	67.38 ± 0.06 ^Ea^	67.60 ± 0.13 ^Da^	68.44 ± 0.24 ^Eb^	69.22 ± 0.09 ^Fc^	68.15 ± 0.08 ^Eb^
	B25	67.82 ± 0.12 ^Fa^	68.96 ± 0.03 ^Eb^	69.92 ± 0.13 ^Fc^	69.26 ± 0.19 ^Fb^	70.36 ± 0.26 ^Fc^
	S25	67.09 ± 0.04 ^Eb^	67.42 ± 0.02 ^Dbc^	67.80 ± 0.10 ^Dc^	67.49 ± 0.11 ^Ebc^	66.46 ± 0.31 ^CDa^
	O25	64.79 ± 0.09 ^Cb^	64.45 ± 0.09 ^Cab^	64.42 ± 0.12 ^Ba^	66.44 ± 0.06 ^Dc^	67.06 ± 0.04 ^Dd^
	M25	66.10 ± 0.04 ^Dd^	64.96 ± 0.13 ^Cb^	65.66 ± 0.17 ^Cc^	64.74 ± 0.03 ^Cb^	66.01 ± 0.09 ^Ccd^
	H25	64.15 ± 0.18 ^Ba^	65.16 ± 0.47 ^Cab^	65.33 ± 0.13 ^Cbc^	66.31 ± 0.24 ^Dcd^	66.51 ± 0.02 ^CDd^
	U25	62.04 ± 0.10 ^Aa^	61.80 ± 0.08 ^Ba^	63.92 ± 0.01 ^Bb^	62.29 ± 0.01 ^Bab^	64.30 ± 0.20 ^Bb^
50% meat replacement	F50	72.92 ± 0.16 ^Cc^	71.77 ± 0.20 ^Bb^	70.75 ± 0.15 ^Ba^	71.92 ± 0.08 ^Bb^	70.37 ± 0.06 ^Ba^
	B50	75.19 ± 0.21 ^Da^	75.95 ± 0.11 ^Fb^	76.96 ± 0.19 ^Fc^	76.91 ± 0.04 ^Gc^	75.55 ± 0.16 ^Eab^
	S50	75.48 ± 0.24 ^D^	75.81 ± 0.08 ^F^	75.56 ± 0.11 ^E^	75.57 ± 0.24 ^E^	75.37 ± 0.24 ^E^
	O50	76.85 ± 0.28 ^Ed^	74.94 ± 0.01 ^Ec^	72.63 ± 0.09 ^Ca^	74.15 ± 0.14 ^Db^	73.76 ± 0.06 ^Db^
	M50	76.12 ± 0.13 ^Db^	75.86 ± 0.01 ^Fb^	76.39 ± 0.06 ^Fb^	76.34 ± 0.06 ^Fb^	72.62 ± 0.37 ^Ca^
	H50	71.16 ± 0.11 ^Ba^	72.33 ± 0.05 ^Cb^	71.04 ± 0.29 ^Ba^	72.67 ± 0.08 ^Cb^	72.45 ± 0.06 ^Cb^
	U50	71.10 ± 0.38 ^Bb^	73.32 ± 0.04 ^Dc^	73.58 ± 0.05 ^Dc^	73.80 ± 0.03 ^Dc^	70.00 ± 0.11 ^Ba^

^A–G^; Means within the same column with different superscripts are different. ^a–d^; Means within the same row with different superscripts are different (Tukey’s post hoc test, *p* < 0.05). C: control, 100% ground pork sausage; F25–F50: sausage with 25–50% fresh fermented oyster mushrooms; B25–B50: sausage with 25–50% blanched fermented oyster mushrooms; S25–S50: sausage with 25–50% steamed fermented oyster mushrooms; O25–O50: sausage with 25–50% oven-treated fermented oyster mushrooms: M25–M50: sausage with 25–50% microwave-treated fermented oyster mushrooms; H25–H50: sausage with 25–50% HHP-treated fermented oyster mushrooms; U25–U50: sausage with 25–50% UV-treated fermented oyster mushrooms.

## Data Availability

The original contributions presented in the study are included in the article, further inquiries can be directed to the corresponding author.
